# HEGPOL: Randomized, placebo controlled, multicenter, double-blind clinical trial to investigate hepatoprotective effects of glycine in the postoperative phase of liver transplantation [ISRCTN69350312]

**DOI:** 10.1186/1471-2482-5-18

**Published:** 2005-08-17

**Authors:** Steffen P Luntz, Kristina Unnebrink, Monika Seibert-Grafe, Hartwig Bunzendahl, Thomas W Kraus, Markus W Büchler, Ernst Klar, Peter Schemmer

**Affiliations:** 1 Coordination Centre for Clinical Trials (KKS), University of Heidelberg, Germany; 2 Department of Surgery, University of Heidelberg, Germany; 3 Department of Surgery, University of North Carolina (UNC) at Chapel Hill, USA; 4 Department of Surgery, University of Rostock, Germany

## Abstract

**Background:**

Kupffer cell-dependent ischemia / reperfusion (I/R) injury after liver transplantation is still of high clinical relevance, as it is strongly associated with primary dysfunction and primary nonfunction of the graft. Glycine, a non-toxic, non-essential amino acid has been conclusively shown in various experiments to prevent both activation of Kupffer cells and reperfusion injury. Based on both experimental and preliminary clinical data this study protocol was designed to further evaluate the early effect of glycine after liver transplantation.

**Methods / design:**

A prospective double-blinded randomized placebo-controlled multicenter study with two parallel groups in a total of 130 liver transplant recipients was designed to assess the effect of multiple intravenous doses of glycine after transplantation. Primary endpoints in hierarchical order are: peak levels of both aspartat-amino-transaminase (AST) and alanine-amino-transaminase (ALT) as surrogates for the progression of liver related injury, as well as both graft and patient survival up to 2 years after transplantation. Furthermore, the effect of glycine on cyclosporine A-induced nephrotoxicity is evaluated.

**Discussion:**

The ongoing clinical trial represents an advanced element of the research chain, along which a scientific hypothesis has to go by, in order to reach the highest level of evidence; a randomized, prospective, controlled double-blinded clinical trial. If the data of this ongoing research project confirm prior findings, glycine would improve the general outcome after liver transplantation.

## Background

### Pathophysiology of liver grafts

The cause of graft failure after transplantation is complex and includes many factors involving organ retrieval, preservation, and the transplantation procedure itself. Important factors include general condition and nutritional status of the donor, cold and warm ischemic times of the graft, operative complications in the recipient, immune status of the recipient and the experience of the surgeon [1,2]. Thus, primary non function (PNF), which is initially determined by reperfusion injury, continues to challenge liver transplantation.

Once activated, Kupffer cells, the resident macrophages in the liver, play a pivotal role for the development of both PNF and primary dysfunction (PDF) [2,3]. Kupffer cell-activation is characterized by an intracellular increase of Ca^2+ ^[4] with a subsequent release of toxic mediators such as proteases, tumor necrosis factor alpha (TNFα), and arachidonic acid derivatives [5,6]. These mediators potentially impair liver function via mechanisms including disturbance of the intrahepatic microcirculation, hypoxia, increased oxygen consumption, and depletion of hepatic glycogen reserves [2].

Fusaoka et al. [7] showed that activated Kupffer cells increase oxygen uptake of the liver after cold storage. This effect is most likely due to Kupffer cell-derived prostaglandine E_2 _(PGE_2_), which stimulates oxygen uptake in hepatocytes and could be involved in early dysfunction of the graft [8]. Indeed, graft survival is impaired after liver transplantation most likely via mechanisms including both hypoxia and increased oxygen consumption of hepatocytes, creating a hypermetabolic state [8]. Activation of Kupffer cells occurs early during organ harvest for transplantation due to in situ organ manipulation, which is inevitable with standard harvesting techniques [1,8,9].

### Glycine

Glycine, a non-toxic, non-essential amino acid is important for the synthesis of many proteins, i.e. creatinine, uric acid, and heme. Under physiological conditions, blood levels of glycine range between 200–400 μmol/L in humans [10,11].

#### Clinical use of glycine

To date various indications for supplementation with glycine have been established, i.e. for total parenteral nutrition, local irrigation during transurethral prostate or urine bladder resections, being a hypotonic solution and having the capacity as an antacidotic agent.

#### Cytoprotective effects of glycine

Addition of amino acids during renal perfusion can protect tubular integrity and can prolong renal function [12]. Weinberg et al. were the first to connect this protective effect with the amino acid glycine [13]. Glycine protects tissue against damage via mechanisims involving proinflammatory mediators, hypoxia reduction, reperfusion enhancement and toxin attenuation in various animal species [10-15].

Glycine inhibits nonlysosomal calcium-dependent proteases and protects hepatocytes against anoxic damage. Ozaki et al. demonstrated that glycine could protect livers in situ from reperfusion damage by minimizing lipid peroxidation [16]. Glycine could stabilize the cell membrane by inhibiting phospholipase A_2 _leading to a reduction of arachidonic acid and eicosanoids which influence hepatic microcirculation [17]. Carolina rinse solution which contains glycine, prevents reperfusion injury to livers in both experimental and human liver transplantation [18]. Intravenous glycine application also prevents Kupffer cell-dependent reperfusion injury in rats [9,19].

#### Kupffer cells

Most recently, a glycine-gated chloride channel (GlyR) has been identified within the membranes of Kupffer cells [10,11,20]. Glycine specifically binds to its receptor. Subsequently, chloride ions enter the cell resulting in the hyperpolarization of the cell membrane and making a Ca^2+ ^influx via voltage dependent Ca^2+^-channels more difficult, which effectively reduces the increase of intracellular Ca^2+ ^[4,9,14,20-22]. As a result, glycine reliably prevents Kupffer cell-dependent reperfusion injury and initial dysfunction of grafts after experimental liver transplantation [1,2,9].

#### Calcineurin inhibitor (CNI)-induced nephrotoxicity

Cyclosporin A (CyA), a calcineurin inhibitor, is widely used as an immunosuppressive agent. Since its introduction in solid organ transplantation, CyA has significantly improved the overall graft survival; however, patients have to maintain therapy for the rest of their lives. Further, this drug is used to treat a variety of autoimmune diseases. Unfortunately, one of the typical side effects of CNIs is dose dependent nephrotoxicity, which is of clinical relevance in up to 30% of patients [23]. Underlying mechanisms most likely include the CyA significant inhibition of respiration in mitochondria isolated from the kidney [24], and thus causing cell damage. Further, CNI-induced nephrotoxicity is characterized by vasoconstriction in kidneys [25], reduced glomerular filtration rate (GFR), and pathological changes such as proximal tubular cell swelling, necrosis, infiltration of macrophages, and interstitial fibrosis [23]. These changes potentially lead to hypoxia-reoxygenation injury involving free radicals. Indeed, a previous study showed that binding of a 2-nitroimidazole hypoxia marker, pimonidazole, in the kidney was increased nearly 3-fold by CyA, indicating marked tissue hypoxia [26].

Most recently, glycine prevented hypoxic and ischemic injury to kidney in rats [26]. This can be explained by the fact that glycine acts as a neurotransmitter with inhibitory effects to the autonomous nervous system, i.e. sympathic nerves [27], decreasing renal nerve firing. This mechanism prevents injury due to CyA [28]. As a result, glycine dilates efferent arterioles and protects cultured proximal tubules from hypoxic injury [29]. Moreover, dietary glycine totally blocked CyA-induced alterations in renal function, such as decreased GFR and pathological changes including cell necrosis and infiltration of macrophages [26,30,31].

#### Study rationale

Both experimental studies and clinical trials [8,9,32-34] have shown that glycine is safe for use in patients [21,22,35-37] and would potentially be beneficial for the treatment of various diseases [10,11]. There is preliminary evidence for beneficial effects of glycine in human liver transplantation [21,22]. However, there is still a lack of solid clinical evidence for these effects of glycine in liver transplantation. The first clinical results with intravenous glycine are very promising [21,22]. Thus to date there is no routine indication for glycine application to liver transplant recipients and no intravenous infusion containing exclusively glycine is commercially available yet.

Based on the beneficial effects of glycine on both liver grafts and kidney function during CyA therapy [26,30,31,38] and its potential application in humans without toxicity, this clinical trial protocol was designed to assess the effects of intravenous glycine in a prospective, double-blind, randomized, placebo-controlled, multicenter study with two parallel groups of liver transplant recipients for the first time in detail.

## Methods / design

After the positive vote of the ethics committee of all involved study sites, enrollment of 130 subjects scheduled for liver transplantation was started in this multicenter, prospective, placebo-controlled, double-blind, randomized clinical trial with two parallel treatment groups (verum / placebo). Only patients who meet all inclusion and exclusion criteria (Table 1) are considered for uptake into the study. Since its initiation in May 2003, about 69 patients have been included until June 2005.

**Table 1:** Criteria of inclusion and exclusion of patients.

Inclusion Criteria	Exclusion Criteria
Patients meeting all of the following criteria are considered for inclusion in the study:	Patients presenting with any of the following are not be included in the trial:
- men and women between 18 and 65 years of age,	- pregnant or nursing women,
- scheduled for first liver transplantation and graft (dead body) already available,	- history of hypersensitivity to glycine or to drugs with a similar chemical structure (amino acids, e.g. serine, threonine, or methionine),
- written informed consent.	- mental conditions rendering the subject incapable to understand the nature, scope, and consequences of the trial,
	- participation in another clinical trial.
	No subject will be enrolled in this study more than once.

**Figure 1:**
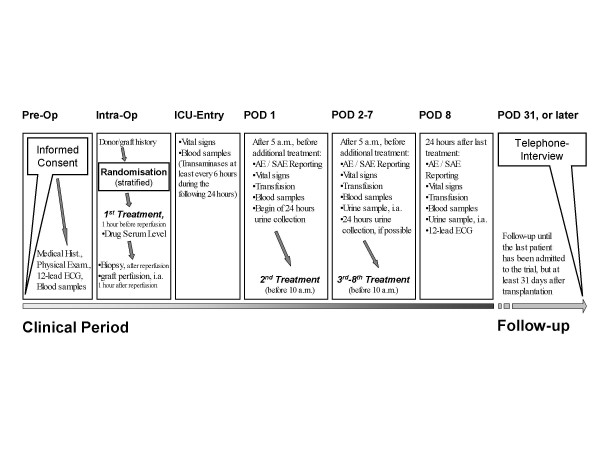
***Scheme depicting the work flow of the study***. After giving informed consent liver transplant recipients are randomized to two parallel groups treated for eight days (day of surgery and the following seven days) using glycine solution or placebo. Follow-up period will be at least 31 days after transplantation.

Patients are treated for a total of 8 consecutive days (day of transplantation and the seven following days). Subsequently, all patients are observed for an additional follow-up period of at least 23 days. Thus every patient is observed for at least one month (Figure 1). In detail, to assess the incidence of a late onset of graft failure based on patients' death or patients' announcement for re-transplantation all patients will be followed-up until one month after the last patient is randomized for this trial. Patients' status will be obtained via phone interview of the patients' general practitioner and / or the responsible transplant consulting office. The Schedule for all study related activities and data collection is listed in Table 2.

**Table 2:** Schedule of study related activities and data collection. The following study related activities are planned for each subject. Blood samples already taken in the routine process at the planned time do not have to be taken twice.

	**Clinical period**						**Follow-up period**
**Study related activity**	**pre-OP**	**OP**	**ICU-entry**	**POD 1**	**POD 2–7**	**POD 8**	

Informed consent	**x**						
Medical history	**x**						
Physical examination	**x**						
Donor and graft history		**x**					
Randomisation		**x**					
Treatment		**x**		**x**	**x**		
**Safety parameters **(measured after 5 a.m. before additional treatment, unless otherwise stipulated)							
12-lead ECG	**x**					**x**	
Vital signs	**x**		**x**	**x**	**x**	**x**	
Blood chemistry	**x**		**x**	**x**	**x**	**x**	
Haematological parameters	**x**		**x**	**x**	**x**	**x**	
Coagulation parameters	**x**		**x**	**x**	**x**	**x**	
CyA (trough concentration)				**x**	**x**	**x**	
Pregnancy testing	**x**						
**Efficacy Parameters **(measured after 5 a.m. before additional treatment, unless otherwise stipulated)							
Biopsy		x	(immediately after rearterialisation)				
Blood flow in portal vein and common hepatic artery		x	(1 hour after reperfusion)				
AST, ALT	x		x#	x#	x	x	
Biltotal, Bildir, Quick, AT III	x		x	x	x	x	
Gly*		x			x (POD3)		
KreaS, HS	x		x	x	x	x	
24-hours-urine (VolU, KreaU)				start	x	x	
Indicators for early onset of graft failure				x	x	x	
Occurrence of late onset of graft failure							X

^#^; AST and ALT will be measured every 4 to 6 hours during the first 24 hours after ICU entry.

*; Samples for glycine plasma level has to be collected immediately after first study drug application during surgery and on POD 3 (after study drug application).

### Objectives and endpoints

The primary objective of this trial is to demonstrate both efficacy and safety of glycine treatment compared to placebo in the postoperative period during the first eight days after liver transplantation and during the long-term follow-up. Secondary objectives are reperfusion injury to the graft and mortality. Furthermore, it will be investigated whether CyA-induced nephrotoxicity can be reduced by glycine.

Both AST and ALT peaks are expected to be surrogates of the extent of reperfusion injury. AST and ALT are the most common parameters for progression of liver related disease. An increase of these transaminases correlates with both primary failure of the organ and graft injury with subsequent organ dysfunction. Thus peak serum level of AST, measured at intensive care unit (ICU) entry and within the first 8 days after transplantation, has been chosen as the most important primary endpoint of this trial. Further primary endpoints ordered hierarchically are ALT peak during the same period and graft survival based on patients' death or announcement for re-transplantation (minimum observation period 31 days). The last reflects the potential clinical benefit for the patient.

Secondary endpoints are the effect of glycine on liver injury based on liver biopsy immediately after re-arterialisation (according to pathological report), total blood flow in portal vein and common hepatic artery 1 hour after reperfusion, graft injury based on both AST and ALT serum levels (area under the curve (AUC)), incidence of early graft failure based on peak of transaminases or clotting factor support, early onset of graft dysfunction based on Quick's value, serum bilirubin (AUC), and CyA-induced nephrotoxicity based on retention parameters during the first eight days after transplantation (AUC).

### Sample size calculation

The sample size calculation is based on the AST peak, the most important primary endpoint in this trial. According to historic data of about 450 liver transplant recipients at the Department of Surgery, University of Heidelberg, a log-normal distribution of the AST peaks is plausible with a standard deviation of 1.01 for the log (AST peak)-values (details are available on request). These data served for the sample size calculation of this trial. A decreased AST-peak by 300 U/L is considered clinically relevant. Thus a sample size of 65 patients per group, i.e. a total of 130 patients, is sufficient to detect differences with a power of 80%, taking into account the planned interim analysis (two-sided t-test for the log (AST peaks), overall level of significance α = 0.05) (nQuery^® ^4.0, EaSt^®^-2000). Details on transformation of the clinically relevant difference on the original scale to a clinically relevant difference on the log scale used for sample size calculation are available on request.

### Randomization and treatment

Here a 1:1 randomization ratio has been chosen. Randomization is stratified for each center and for the duration of cold ischemia (≤ 10 hrs or >10 hrs). The study medication is produced, labeled, and packed by a Clinical Pharmacy Department. The guideline for good manufacturing practice (GMP) is adhered to. The intravenous medication for the verum group contains 250 ml glycine solution (4.4%; 11 g glycine, dissolved in aqua ad injectione). In contrast the placebo consisted of 250 mL of the vehicle (aqua ad injectionem) have to be infused to patients.

All patients receive their first study medication via a central venous line during liver transplantation one hour prior to reperfusion. During the following week, on post operative days (POD) 1 to 7, the study medication is infused once per day in the morning, after taking blood and urine samples for safety and efficacy parameters. A last visit is scheduled for POD 8 to investigate the patients' clinical status and to collect all parameters for efficacy and safety. To detect late graft failures all patients are observed after the initial first eight days until the last patient is enrolled in this trial. These patients will then be observed for at least 31 days after transplantation. A late graft failure is defined as patients' death or announcement for re-transplantation.

If it is medically imperative to know the patient's treatment, emergency envelopes contain the information on the subject's study medication. Those are to be opened only under emergency circumstances. During the trial a concomitant treatment may be given at the discretion of the investigator, if these are considered necessary for the subject's welfare.

All patients routinely receive standard immunosuppressive therapy. The initial dose of CyA is 2 × 2 mg/kg body weight during the first 24 hrs. Subsequently, daily doses of CyA are adapted to the actual CyA serum level which is measured in the blood samples taken in the morning of POD 1 to 8. The trough concentration should be between 200–250 μg/L during the first month after liver transplantation. In case of renal insufficiency FK506 can be used alternatively.

The amount of coagulation factors and AT III, red blood cells, and fresh frozen plasma given after transplantation are documented until the morning of POD 8.

### Adverse events

All adverse events (AE) are recorded. Events related to the initial diagnosis for liver transplantation, to the transplantation procedure itself, or problems associated with routine procedures after transplantation, i.e. liver biopsy, are not to be noted as AE or serious adverse event (SAE) unless the investigator deems the events to be a cause of the study drug. All SAE potentially associated with the application of study medication must be documented on a "Serious Adverse Event" form which has to sent to the principal investigator (LKP, according to German Drug Law) within 24 hrs or latest on the following working day. The LKP ensures that SAE are reported to the safety board, ethics committees, and to further investigators, if applicable.

### Quality assurance

The study is performed according to the principles of the ICH-GCP guidelines [39] and the ethical principles according to the current revision of the Declaration of Helsinki [40] and local legal and regulatory requirements. The trial is monitored by the KKS Heidelberg according to Standard Operation Procedures (SOP) that are based on ICH-GCP guidelines.

An independent safety board monitors closely the proper conduct of the trial and all SAE reports to ensure the safety of the subjects during the course of the study.

### Statistics and data management

All findings including both the clinical and laboratory findings are documented in the subject's case report form (CRF). All data are entered in a database as recorded. To ensure highest data quality a double data entry is performed. All missing data or inconsistencies are reported back to the center(s) and clarified by the responsible investigator. If no further corrections of the database are to be made it will be declared closed and used for statistical analysis.

The primary endpoints of the trial are peak serum levels of both AST and ALT measured within the first 8 days after transplantation, and graft survival based on patients death or announcement for re-transplantation.

Therefore the statistical hypotheses to be tested are as follows:

H0: AST_glycine _= AST_placebo _vs. H1: AST_glycine _≠ AST_placebo_,     (1)

H0: ALT_glycine _= ALT_placebo _vs. H1: ALT_glycine _≠ ALT_placebo_,     (2)

H0: S_glycine _= S_placebo _vs. H1: S_glycine _≠ S_placebo_,    (3)

While AST_glycine _and AST_placebo _represent the distribution of AST peaks in the glycine and placebo group, respectively, ALT_glycine _and ALT_placebo _represent the distribution of ALT peaks in the two groups, and S_glycine _and S_placebo _represent the survival functions of the two groups regarding graft survival.

The statistical hypotheses formulated above will be tested, each at the level of significance α (see below), in the strict order (1), (2), (3), i.e. a statistical testing procedure with a priori ordered hypotheses is applied [41].

All analyses are done according to the principle of intention-to-treat. Additional analyses (per protocol population, sensitivity analyses) will be described in the statistical analysis plan in more detail before closure of the database.

In the interim analysis only safety parameters and the primary endpoints AST peak and ALT peak will be evaluated. Data for the third primary endpoint (graft survival) are collected only at the end of the trial. The interim analysis is performed according to the group sequential design of O'Brien and Fleming [42]. The overall level of significance is α = 0.05, i.e. the level of significance for the interim analysis is α 1 = 0.0035, the level of significance for the final analysis is α2 = 0.0488.

If a patient dies or a re-transplantation has to be performed during the clinical period, serum measurements are not available. As this is the worst possible outcome, patients will be allocated the worst ranks for the respective (non-parametric) analyses.

Sensitivity analyses regarding the methods for dealing with missing values will be performed. Further details regarding the handling of missing data will be laid down in the statistical analysis plan that will be completed before unblinding of the data.

## Discussion

This ongoing clinical trial particularly demonstrates how a scientific hypothesis has been developed from bench to bedside, with classical investigations along the value chain of translational research from early in vitro work to in vivo experiments and finally from single case observations to the highest level of evidence, a randomized, prospective, controlled, double-blinded clinical trial. If previous findings are confirmed by the data of this ongoing clinical trial, glycine would improve the overall outcome after liver transplantation.

## Competing interests

The author(s) declare that they have no competing interests.

## Authors' contributions

SPL participated in the design of the study, developed essential study documents and acts as coordinating investigator (Leiter der Klinischen Prüfung (LKP) according to German drug law). KU participated in the design of the study, developed the statistical part of the study protocol and the statistical analysis plan. MSG performed quality review to assure adherence to current guidelines and laws. HB, TWK, MWB, and EK supported the design of the study with their knowledge and experience. PS conceived and designed the study based on his own preclinical and clinical results. Further he designed and conducts the study as the main investigator. PS and SPL wrote the manuscript. All authors read and approved the final manuscript.

## Pre-publication history

The pre-publication history for this paper can be accessed here:


